# Can grapheme-color synesthesia be induced by hypnosis?

**DOI:** 10.3389/fnhum.2014.00220

**Published:** 2014-04-28

**Authors:** Hazel P. Anderson, Anil K. Seth, Zoltan Dienes, Jamie Ward

**Affiliations:** ^1^School of Psychology, University of SussexBrighton, UK; ^2^Sackler Centre for Consciousness Science, University of SussexBrighton, UK; ^3^School of Engineering and Informatics, University of SussexBrighton, UK

**Keywords:** hypnosis, synaesthesia/synesthesia, colour/color, embedded figures test, consciousness, mental imagery

## Abstract

Grapheme-color synesthesia is a perceptual experience where graphemes, letters or words evoke a specific color, which are experienced either as spatially coincident with the grapheme inducer (projector sub-type) or elsewhere, perhaps without a definite spatial location (associator sub-type). Here, we address the question of whether synesthesia can be rapidly produced using a hypnotic color suggestion to examine the possibility of “hypnotic synesthesia”, i.e., subjectively experienced color hallucinations similar to those experienced by projector synesthetes. We assess the efficacy of this intervention using an “embedded figures” test, in which participants are required to detect a shape (e.g., a square) composed of local graphemic elements. For grapheme-color synesthetes, better performance on the task has been linked to a higher proportion of graphemes perceived as colored. We found no performance benefits on this test when using a hypnotic suggestion, as compared to a no-suggestion control condition. The same result was found when participants were separated according to the degree to which they were susceptible to the suggestion (number of colored trials perceived). However, we found a relationship between accuracy and subjective reports of color in those participants who reported a large proportion of colored trials: trials in which the embedded figure was accurately recognized (relative to trials in which it was not) were associated with reports of more intense colors occupying a greater spatial extent. Collectively, this implies that hypnotic color was only perceived after shape detection rather than aiding in shape detection via color-based perceptual grouping. The results suggest that hypnotically induced colors are not directly comparable to synesthetic ones.

## Introduction

Individuals with grapheme-color synesthesia experience a color (concurrent) when viewing particular letters, numbers or grammatical symbol (inducer). The triggered color experience is automatic (Mattingley et al., 2001) and the concurrent color is consistent over time (Simner et al., 2005). The developmental form of synesthesia emerges early in life (Simner et al., 2006), is associated with genetic differences (Tomson et al., 2011), and also structural and functional differences within the brain (Hubbard et al., 2005; Rich et al., 2006; Rouw and Scholte, 2007; Weiss and Fink, 2009). Early development may not be the only pathway for the emergence of synesthetic experiences (unless, of course, one chooses *a priori* to limit the term “synesthesia” to particular causal mechanisms). It has long been known that synesthesia can be acquired as a result of sensory loss (Armel and Ramachandran, 1999) or temporarily after taking hallucinogenic drugs (Sinke et al., 2012; Luke and Terhune, 2013). More recently, synesthesia has been reported to arise after brain damage (Ro et al., 2007) and it has also been suggested that synesthesia may arise, in blind individuals, after expertise with sensory substitution technology (Ward and Wright, 2012; see also Ward and Meijer, 2010; Farina, 2013). Finally, it has been claimed that synesthesia can be induced by hypnosis in hypnotically suggestible individuals (Cohen Kadosh et al., 2009). In the present study we re-examine this claim using an “embedded figures” test that has been widely used in grapheme-color synesthetes. The benefit of this task is that evidence for synesthesia type behavior would be measured through task improvement, rather than through deficits, which are easier to produce through task compliance. The issue is of theoretical importance because positive findings would suggest that synesthesia can (at least in some circumstances) arise from purely functional—rather than structural—brain changes (Grossenbacher and Lovelace, 2001) and, moreover, that hypnosis can create novel perceptual abilities.

Hypnosis is able to alter the phenomenological properties of participants’ subjective experience (Kihlstrom, 2013; Oakley and Halligan, 2013). The process of hypnosis can be divided into induction and suggestion stages. In the induction stage, a putative “hypnotic state” is induced, or expectations for experiences are heightened, or the subject is simply alerted that the context is appropriate for a certain sort of response (e.g., Oakley and Halligan, 2009; Kirsch, 2011); in the second stage, suggestions are given to the participant to experience a (potentially) wide range of physical and perceptual experiences. There is considerable variability both in individual hypnotic susceptibility (Bowers, 1998) and in the range of experiences that can be induced. Importantly, perceptual hallucinations (e.g., hearing music) can be induced in many participants (Bowers, 1998), providing a potential link to synesthesia.

Although there is general consensus that hypnosis can alter a participant’s subjective experience and this can then cause behavioral changes (Kihlstrom, 2013), the neural processes underlying the functional changes corresponding to hypnotically induced perception remain poorly understood. One class of theories postulates that highly hypnotizable people can perform tasks when hypnotized that they could not do otherwise; for example, distort perception so that they actually can see non-existent objects in a way they could not imagine (e.g., Brown and Oakley, 2004) or fail to perceive stimuli that would otherwise impinge on their awareness (e.g., pain, Hilgard, 1986). Another class of theories postulates that highly hypnotizable people cannot do anything hypnotically that they could not do anyway (e.g., Sarbin and Coe, 1972; Spanos, 1986). One way of characterizing the latter class of theory is in terms of “cold control” (Dienes, 2012), which postulates that the defining feature of acting hypnotically is simply the incorrect meta-cognition that one is not intending the (motor or cognitive) action. For example, hypnotic hallucination of an object on this account is imagining the object, but without realizing that one is deliberately creating a visual representation: It appears to occur from other causes and thus appears as perception (Dienes and Perner, 2007). That is, according to cold control, hypnotic responding involves no new abilities, just the sense that an action is happening by itself. The two classes of theory can be tested by using suggestions for abilities not already possessed by subjects: If the subject gains abilities they did not have, the second class of theory is refuted. As we will argue, suggestions for synesthetic experiences can serve this function. Synesthetic experience has some perception-like qualities that may enable enhanced performance on some tasks (Ramachandran and Hubbard, 2001) so the question arises whether hypnotically-induced synesthetic experience is more perception-like than imagined synesthetic experience.

The generation of synesthetic experience appears automatic, and automatic processes are partially defined by the difficulty in controlling them. So the question is raised whether control of synesthetic experience might be greater hypnotically rather than non-hypnotically. One developmental form of grapheme-color synesthesia has been temporarily reduced through hypnotic suggestion. Terhune et al. (2010) abolished phenomenological synesthetic experiences in a participant AR, a synesthete who experiences colors when viewing faces. She had to name the colors of face stimuli which were presented in either congruent or incongruent colors. Stroop-like interference effects were evident in comparison to controls in both reaction times (RTs) and event related potentials (ERPs) at baseline, however, after a post hypnotic suggestion these were no longer evident. This indicates the relevance of synesthesia to testing theories of hypnosis, as well as for hypnosis for testing theories of synesthesia. We will consider the converse case to that of Terhune et al., namely, of hypnotically suggesting a type of synesthesia in people who did not previously experience it.

To create a “hypnotic synesthesia” one can use the suggestion that when seeing (for example) the letter A the participant will see a special red color on the page. Supporting this idea, the phenomenological perception of color (or no color) has been manipulated using suggestion to add or drain color from patterned stimuli (Kosslyn et al., 2000). High but not low susceptible participants all reported being able to see gray-scale stimuli as colored, and colored stimuli as gray-scale when given hypnotic suggestion to do so. Further, positron emission tomography (PET) indicated changes in cerebral blood flow for left and right fusiform areas (perhaps corresponding to human V4) when hypnotic induction was used; however, non-hypnotically imagining the color changes produced significant changes only in right fusiform cortex. Hypnosis appeared to influence activity in color-sensitive areas of the brain in a way imagination alone could not.

The Kosslyn et al. (2000) study highlights the issue of demand characteristics in hypnosis research, and the tendency of subjects to either “hold back” or try harder in different conditions to produce the pattern of results they perceive as desired (Orne, 1962; Spanos, 1986). Specifically, the suggestion was more strongly worded in the hypnosis rather than the imagination condition in order that subjects would not confuse the two conditions and “slip into trance” in the imagination condition. Kirsch et al. (2008) presented the identical color adding or draining suggestions with or without hypnotic induction, and obtained substantial and near equivalent changes in color perception in both conditions. (Further, subjects rated themselves as clearly not hypnotized in the non-hypnotic condition, indicating that slipping into trance was not a problem.) Thus, when demand characteristics were more nearly equalized the difference between hypnosis and non-hypnosis in subjective experience greatly diminished. Further, McGeown et al. (2012) showed that similar activation in visual areas was produced in both the hypnotic and non-hypnotic conditions. That is, as cold control theory predicts, hypnotic hallucination with the suggestions used by Kosslyn et al. (2000) may involve the same visual abilities as imagination, with the difference being purely metacognitive (Dienes, 2012).

There has been one previous attempt to hypnotically induce grapheme-color synesthesia (Cohen Kadosh et al., 2009). Cohen Kadosh et al. assigned colors to digits either through post-hypnotic suggestion or learnt association (e.g., 5 = green). Participants were required to search for an achromatic (black) grapheme against a colored background. The results showed that search was impaired when the suggested color was congruent with the background. However, this result reflects a deterioration in performance which is easier to simulate (explicitly or implicitly) than an improvement. Cohen Kadosh et al. requested control groups to associate the colors with the digits in non-hypnotic contexts; the non-hypnotic request had no effect on the search task. However, a non-hypnotic request carries different demand characteristics from a hypnotic or post-hypnotic suggestion. It is also unclear from this study whether the performance on the task does indeed resemble that found in developmental grapheme-color synesthesia. To our knowledge only one developmental grapheme-color synesthete has been tested on a version of this task (Smilek et al., 2001). While performance of this synesthete showed the same trend as the “hypnotic synesthetes” (i.e., worse performance on congruent trials, about 20%, compared to incongruent trials, about 90%), they were far from equivalent in other respects. Perhaps surprisingly, the manipulation had only a mild effect on the synesthete (88% correct for congruent and 96% for incongruent conditions) but a drastic effect on the hypnotized non-synesthetes. Although direct comparison of proportion correct trials is difficult due to differences in the task, the comparison is striking as the synesthete completed a more difficult task than the non-synesthetes, being required to provide a specific grid location for the target grapheme amongst distracters. Furthermore, although behavioral similarities between developmental and hypnotic synesthetes are informative of cognitive processes, they don’t provide detail of the phenomenological experience of the participant, an aspect which requires more attention in hypnotic synesthesia research as well as more generally in neuroscience (Lifshitz et al., 2013).

In the present study, we re-examine whether hypnotically induced synesthetic colors can lead to facilitated performance on the Embedded Figures Test. Although the test itself (and the interpretation of the results) is not without controversy, it has the advantage of predicting that synesthetic (or hypnotically hallucinated) colors should facilitate rather than impair performance on a difficult task, as in the previous study. Moreover, the test has been utilized in several previous studies involving grapheme-color synesthetes providing useful benchmark comparisons. Ramachandran and Hubbard (2001) showed synesthetes arrays comprising of different graphemes (e.g., 5 s and 2 s) such that one of the graphemes could be grouped together to form a shape (e.g., a triangle made of 2 s).^1^ The task was to identify the global shape, from four alternatives, given a limited viewing time of 1 s. Ramachandran and Hubbard (2001) found their two synesthetes to be significantly more accurate in identifying the embedded shape than controls. They later called this effect “pop-out” (Hubbard et al., 2006).

The effect was partially replicated by Hubbard et al. (2005) who noted that the “pop out” effect was not as great as would be expected for true color. Rothen and Meier (2009) however did not find an accuracy advantage for synesthetes in comparison to controls for the same task. Ward et al. (2010) partially supported the original findings with their larger scale replication study involving a sample of 36 synesthetes. Synesthetes’ accuracy at detecting embedded shapes was significantly higher than controls, though detection rates (41% for synesthetes) remained significantly below that corresponding to true “pop out”. In this study, participants were also required to rate the phenomenal vividness of the synesthetic color and to indicate what percentage of the digits appeared as colored. Importantly, the greatest performance benefits were found for those synesthetes who experienced a large proportion of the array as colored. This was interpreted by proposing that synesthetic colors may facilitate local grouping within a spatial window of attention but that synesthetic colors do not enable pre-attentive pop-out. The latter interpretation may explain why other studies, based on more standard visual search paradigms, have often failed to find any benefit of synesthetic colors in detecting a target achromatic singleton grapheme (Edquist et al., 2006; Sagiv et al., 2006). That is, synesthesia may assist local grouping of elements on the basis of color (facilitating the embedded-figures test) but synesthetic color may not enable pre-attentive pop-out (on more standard visual search).

As noted by Ward et al. (2010), grapheme-color synesthetes differ in the extent to which they perceive (or notice) their synesthetic colors during this task. Synesthetes classed as “projectors”, i.e., who report their colors in the spatial location of the grapheme, were more likely to report colors (and showed a trend to do better overall on the task). The reasons for this are not completely understood (Ward et al., 2007, 2010). Nevertheless, for the present purposes we decided to optimize the chances of obtaining a significant result by instructing our highly hypnotizable participants to project colors onto the array of graphemes. If hypnotic suggestions can create grapheme-color synesthesia then hypnotically hallucinated colors will facilitate performance on this task (relative to a no-suggestion control condition). We also ask, if hypnotic grapheme-color synesthesia can be induced, are the perceptual reports similar to those of natural synesthetes in regards to the vividness and percentage of colored digits?

## Method

A counterbalanced two (condition; hypnotic suggestion vs. no-suggestion) by four (duration; 1, 2, 3 and 4 s) within subjects design was used.

## Participants

Fourteen participants aged 18–42 (*M* = 23.2, SD = 6.3) were recruited through the University of Sussex Hypnotic Susceptibility Register. Each had previously been screened using the Waterloo-Stanford Group Scale of Susceptibility, form C (Bowers, 1998) with a score of 8 or higher being used to classify them as highly susceptible. This corresponds to the upper 10% of people screened. Scores ranged from 8–11 (*M* = 9, SD = 1.04). Each was paid £5 for participation, the whole experiment lasting approximately 1 h. No participant reported having any type of synesthesia, this was asked prior to testing. The study was granted clearance from the University of Sussex ethics committee.

## Materials

The embedded figures stimuli consisted of four shapes (squares, rectangles, triangles and diamonds) of number 2 s embedded in an array of 5 s taken from the Ward et al. (2010) study. Each shape was made from 6 to 10 target 2 s surrounded by 41 distracter 5 s, all of which were in black font. Participants sat approximately 85 cm from a 15″ LCD monitor with 60 Hz refresh rate. The shape location differed across trials, not always appearing in the center. E-prime 2.0 software was used to run the experiment.

## Procedure

The experiment was repeated twice within each session, counterbalanced so that half of the subjects completed the hypnosis condition first, and the other half the control. Participants were not informed that the study would include hypnosis until just prior to the hypnosis condition to avoid “hold back” (Stam and Spanos, 1980) where participants may unconsciously perform poorer in the baseline condition than they were capable of.

**Figure 1:**
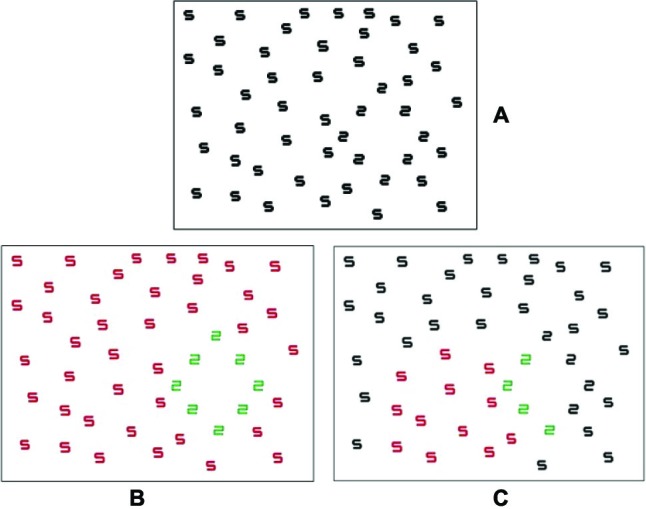
**An example stimulus **(A)** as presented (in black font), **(B)** a schematic assuming presentation in color, and **(C)** a partially colored version in line with the phenomenological reports of projector synesthetes**. Note that stimuli were never presented in color during the experiment.

The experiment consisted of four blocks. For half of the participants the block order was ascending (1 to 4 s duration), for the other it was descending (4 to 1 s). In the hypnosis condition, participants received a brief induction (where the participant was asked to become relaxed and counted down into a deep hypnotic state) before a hypnotic suggestion to see green for 2 and red for 5 on the monitor where the digit was, very much like a projector synesthete. Prior to each hypnosis block, the suggestion was reinforced by requiring the participant to focus on an individual stimulus digit and attempt to enhance the color. This was done for both the 2 and 5. If they did not see any color, they were asked to attempt to visualize it as colored as best they could. Figure 1 shows how the stimulus would be colored if color experiences phenomenologically similar to those of natural projector synesthetes were evoked using hypnotic suggestion. When completing the control block, no specific instructions were given on how to complete the task.

## Suggestion for hypnotic grapheme-color synesthesia

“Now you will see on the computer screen many 2 s and 5 s. Whenever you see the digit five you will experience it as having a special red color. Similarly, whenever you see the digit two you will experience it as having a special green color. I want you to make the special color as vivid as possible, actually see the color there. Soon you will be presented with a screen containing both 5’s and 2’s. You will be able to see the 5’s as vividly red and the 2’s as vividly green. On each trial there will be a green shape made up of 2’s. You must select the shape you see. The trials will be presented for 1, 2, 3 or 4 s. Is that clear? Ok we can start”.

**Figure 2:**
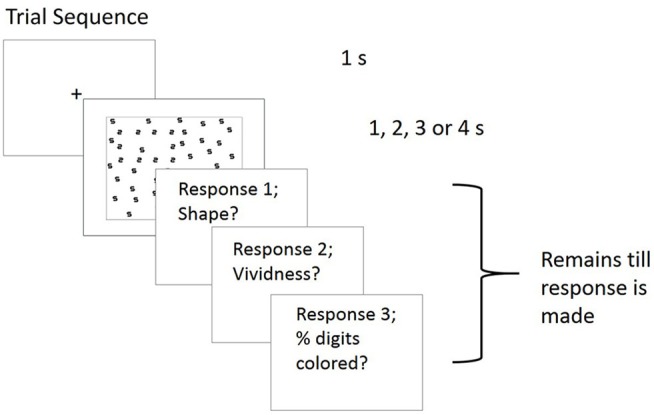
**Experiment trial sequence.** For response 1, shapes (square, rectangle, diamond and triangle) were presented with response keys underneath (keys 1–4). Response 2, was measured using a six point scale (1 = no color, 6 = very vivid color) followed by response 3, the percentage of digits within the array which were colored which was typed by the participant.

An example trial was given at the start of each block where they were reminded to find the shape made of 2 s, and the responses required. Each trial was preceded by a central fixation cross for 1 s. The stimulus was then displayed for 1, 2, 3 or 4 s depending on the block, followed by a blank screen containing instructions to respond using a four-alternative forced choice to indicate the shape (square, rectangle, diamond or triangle). Following this, they were asked to rate their subjective experience of color during the trial display on two scales. Specifically, they were asked to rate the vividness of any perceived colors (1 = no color, 6 = very vivid color) followed by the percentage of digits within the array which were colored. The fixation cross then appeared to signal the start of the next trial, see Figure 2 for trial sequence. Accuracy was emphasized and participants were aware that the proceeding trial did not begin until a response had been made for the current trial. On completing the hypnosis condition, the suggestion was removed (by stating that numbers no longer had any special colors, and appear as they did before any suggestion was given) then the participant was counted out of hypnosis.

## Results

### Accuracy of target detection

**Figure 3:**
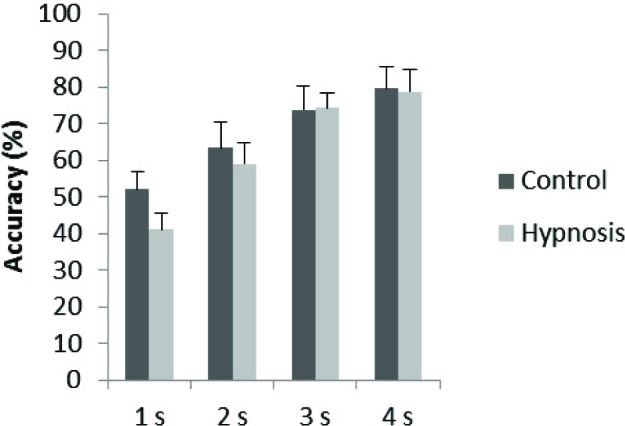
**Shape detection accuracy for control and hypnosis conditions across stimulus durations (1–4 s).** Error bars represent 1 SEM.

The accuracy data were measured using percentage accuracy and were analyzed as a 2 × 4 repeated measures ANOVA contrasting condition (presence/absence of hypnotic suggestion) and duration of stimulus (1–4 s). The main effect of duration was significant (*F*_(1.78,  22.13)_ = 45.16, *p* < 0.001) with pairwise comparisons using Bonferroni adjustment showing significant differences between all durations (*p* < 0.001) other than between 3 and 4 s (*p* = 0.13) due to accuracy improving when the arrays were presented for longer durations (1 s *M* = 46.6%, MSE = 3.0; s *M* = 61.1%, MSE = 4.8; 3 s *M* = 74.0%, MSE = 4.5; 4 s *M* = 79.1%, MSE = 5.3). The main effect of condition was not significant (*F*_(1, 13)_ = 0.62, *p* = 0.45): accuracy for the control condition (*M* = 67.2%, MSE = 5.5) was similar to that in the hypnosis condition (*M* = 63.2%, MSE = 3.9). The interaction was also not significant, with accuracy being similar between control and hypnosis conditions for each duration (*F*_(3, 39)_ = 0.88, *p* = 0.46). This data is depicted in Figure 3. To determine whether the lack of main effect of condition reflected insensitive data, or supported a null hypothesis, we used a Bayes factor analysis (Dienes, 2011). Whereas significance testing only allows the null hypothesis to be rejected, Bayes factor analysis also allows the null hypothesis to be supported (Kass and Raftery, 1995). If the Bayes factor is less than 1/3 there is substantial evidence for the null over the specified alternative; if greater than 3, substantial evidence for the alternative; otherwise the data are insensitive in distinguishing the two hypotheses. Ward et al. (2010) found that synesthetes were better than normal on this task by 10%; thus this was used as the standard deviation of a half-normal to represent an alternative hypothesis that the hypnosis suggestion created a genuine synesthesia (as per the guidelines in Dienes, 2011 Appendix). With an actual mean difference of −4% (SE = 5.1%), the Bayes factor is 0.28, i.e., there was substantial evidence for the null hypothesis, that there is no difference between percentage accuracy for the hypnosis vs. control condition.

### Phenomenological reports

We next considered the extent to which the participants experienced colors during the task. Table 1 shows the number of trials in which color was perceived, the average vividness of colors reported by each participant, and the percentage of graphemes in the array that were perceived as colored. These data are reported only for the hypnotic suggestion condition. Twelve out of the 14 participants experienced some color during the hypnosis condition. For those who did experience color, the proportion of digits and intensity in which they saw color was extremely variable across participants.

**Table 1:** **Summary of subjective color experiences for each participant for the hypnosis condition only in descending order from the participant who experienced color for the most trials to least**.

**Part. Number**	**% Colored Trials**	**% of Graphemes Perceived as Colored (all trials)**				**Average Intensity (all trials)**			
		**1 s**	**2 s**	**3 s**	**4 s**	**1 s**	**2 s**	**3 s**	**4 s**
**11**	100	52	57	49	41	3.96	3.96	3.68	3.79
**2**	99	63	80	81	81	3.75	5.04	5.04	5.46
**8**	97	24	37	39	41	2.64	3.57	3.89	4.25
**17**	97	90	60	56	58	4.32	3.89	3.00	3.07
**6**	96	37	34	38	42	3.07	3.29	3.32	3.89
**12**	83	47	61	67	61	3.50	3.89	4.61	3.64
**7**	81	7	3	6	5	1.50	1.25	1.75	1.50
**1**	51	29	27	10	1	2.61	2.32	1.54	1.14
**16**	29	3	7	3	5	1.21	1.39	1.25	1.32
**15**	14	1	0	2	10	1.11	1.04	1.11	1.29
**3**	12	7	0	0	0	1.43	1.00	1.00	1.00
**14**	1	0	0	0	0	1.00	1.04	1.11	1.00
**9**	0	0	0	0	0	1.00	1.00	1.00	1.00
**10**	0	0	0	0	0	1.00	1.00	1.00	1.00

*The table shows the percentage of trials where a color was reported, average intensity (1 = no color, 6 = vivid color) and percentage of graphemes within the array perceived as colored*.

A one-way ANOVA comparing intensity ratings across stimulus duration was not significant (*F*_(1.45, 18.87)_ = 0.19, *p* = 0.76): that is, intensity ratings were similar across all durations (1 s *M* = 2.29, MSE = 0.33; 2 s *M* = 2.41, MSE = 0.39; 3 s *M* = 2.38, MSE = 0.40; 4 s *M* = 2.38, MSE = 0.42). (Note that Ward et al., 2010 reported a similar average vividness rating, 3, for genuine synesthetes.) Similarly, a one-way ANOVA comparing percentage of graphemes perceived as colored across stimulus duration was not significant (*F*_(1.52, 19.73)_ = 0.11, *p* = 0.84) with comparable percentage of grapheme appearing as colored across all durations (1 s *M* = 25.72%, MSE = 7.64; 2 s *M* = 26.25%, MSE = 7.68; 3 s *M* = 24.99%, MSE = 7.72; 4 s *M* = 24.75%, MSE = 7.57) (Ward et al., 2010 reported a similar percentage, 30%, for genuine synesthetes). The duration of stimulus presentation did not substantially affect the phenomenological experience of color.

### Relationship between accuracy and phenomenological reports of color

In order to better understand the role, if any, that color experience played in shape detection the relationship between accuracy and the phenomenological color reports was explored.

For these analyses, the participants were divided by a median split according to the number of trials in which they reported color experiences thereby creating two groups: many or few color responses to the color suggestion.

**Figure 4:**
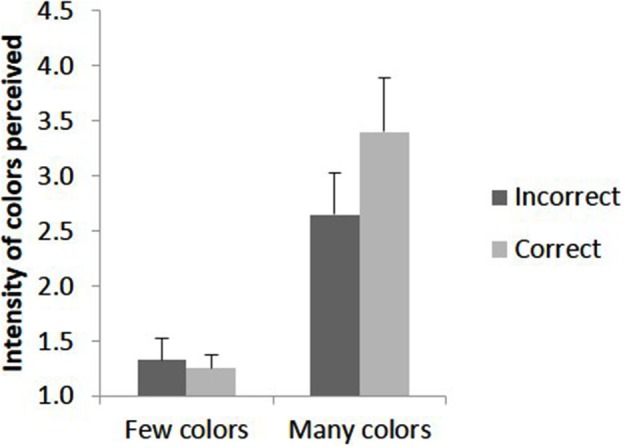
**The mean intensity of colors (1 = no color, 6 = vivid color) reported for trials in which the embedded figure was correctly or incorrectly detected dependent on whether the participant saw many colors or few colors**. Error bars represent 1 SEM.

There is evidence for a relationship between accuracy and phenomenological report when trials are grouped by accuracy. Participant 10 who never experienced colors had 100% accuracy for the 4 s duration block preventing comparison between correct and incorrect trials; after excluding this participant there remained six participants in the group who experienced few colors and seven in the group who experienced many colors. Using phenomenological ratings (mean intensity, mean number of graphemes perceived as colored) as the dependent variables a 2 × 2 × 4 ANOVA was conducted contrasting group (many vs. few color-responses), accuracy (correct vs. incorrect trials) and duration (4 levels). The data are summarized in Figure 3. For intensity, there was a significant interaction of group X accuracy (*F*_(1, 11)_ = 5.09, *p* = 0.045). All other main effects and interactions were not significant. The interaction was analyzed further by simple effects of accuracy for each group. For the group who experienced few colors, the effect of accuracy was not significant (*F*_(1, 5)_ = 0.016, *p* = 0.71) however for the group who experienced many colors this main effect was significant (*F*_(1, 6)_ = 6.32, *p* = 0.046) as more vivid colors were reported for accurate compared to inaccurate trials. This is summarized in Figure 4.

**Figure 5:**
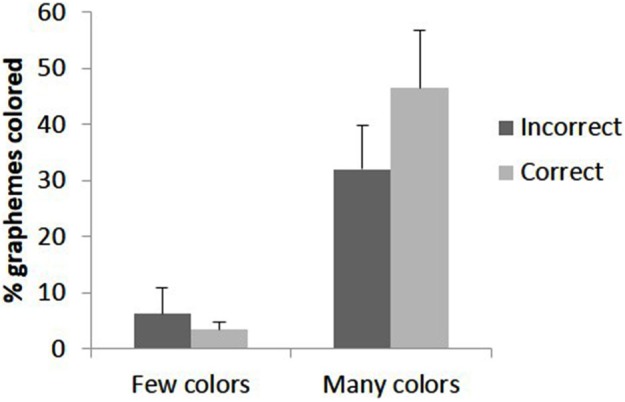
**The percentage of graphemes within the array being reported as colored for trials in which the embedded figure was correctly or incorrectly perceived**. Error bars represent 1 SEM.

For the percentage of graphemes perceived as colored, there was again a significant interaction of group X accuracy (*F*_(1, 11)_ = 5.80, *p* = 0.035). The other main effects and interactions were not significant. A simple effects analysis indicated that for the group who experienced few colors, the effect of accuracy was not significant (*F*_(1, 5)_ = 0.51, *p* = 0.51) whereas for the group who experienced many colors it was significant (*F*_(1, 6)_ = 6.51, *p* = 0.043) with more colors being reported for accurate opposed to inaccurate trials. The data are summarized in Figure 5. Together, the results from Figures 4 and 5 show that participants who saw many colors had more intense and widespread phenomenological color experiences for trials in which they correctly compared to incorrectly identified the embedded shape.

**Figure 6:**
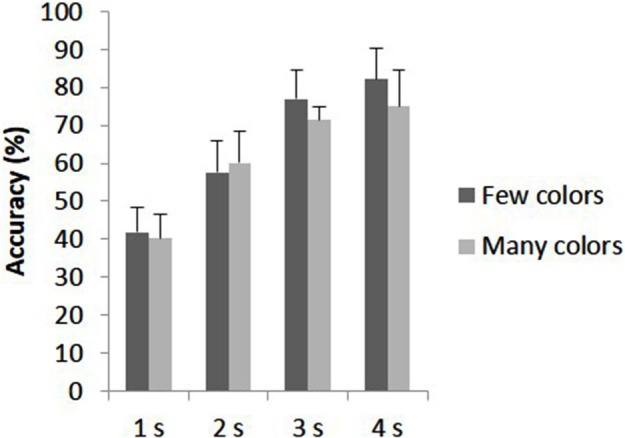
**Shape detection accuracy for lots and little color responders during the hypnosis condition across stimulus durations (1–4 s)**. Error bars represent 1 SEM.

Although the different groups report different levels of color intensity and disparity on correct vs. incorrect trials, the overall number of correct trials didn’t differ according to these groups. A 2 × 4 repeated measures ANOVA with percentage correct as the dependent variable contrasting group (experiencing few vs. many colors) and duration of stimulus (1–4 s) was conducted, considering only the hypnotic-suggestion condition. This data is summarized in Figure 6. The main effect of duration was significant (*F*_(1.67, 20.09)_ = 18.88, *p* < 0.001) with accuracy improving as stimulus duration increased. The interaction between group and stimulus durations was not significant (*F*_(1.67, 20.09)_ = 0.31, *p* = 0.70). Importantly, the difference in accuracy for the hypnosis block between those who experienced many (*M* = 61.7%, MSE = 5.7) and few (*M* = 64.7%, MSE = 5.7) color responses to the synesthesia suggestion was not significant (*F*_(1, 12)_ = 0.13, *p* = 0.72). To interpret the non-significant result, a Bayes Factor analysis was conducted. Again a half-normal distribution was chosen to test the alternative hypothesis that the group who experienced many vs. few colors performed better, representing a real synesthesia like behavioral advantage in those who had phenomenological experience of the colors. As Ward et al. (2010) measured a 10% accuracy advantage for synesthetes, this was used as the SD. With a mean difference of −3, and MSE of 9.5 the resulting Bayes Factor was 0.58 which is between 1/3 and 3 and therefore indicates insensitive data.

## Discussion

We aimed to determine whether hypnotic synesthesia was similar either behaviorally or phenomenologically to developmental synesthesia through measuring accuracy and color experience during an embedded figures task with and without hypnotic suggestion. Under the hypnotic suggestion the phenomenology of the participants was similar to that documented for synesthetes. Specifically, they tended not to perceive the entire array of graphemes as colored and the subjective intensity ratings were similar to those reported by synesthetes. However, the behavioral advantage previously found for synesthetes was not found under hypnotic suggestions, even when one only considers those subjects who responded strongly to the specific suggestions. Further analyses (using Bayes factors) suggested that this was not merely due to a lack of sensitivity. As such, our conclusion is that hypnotically induced grapheme-color experiences are not equivalent to those in developmental synesthesia.

On first impressions, this result seems at odds with Cohen Kadosh et al. (2009), who found that hypnotically suggested synesthesia results in similar performance to developmental synesthetes, showing a deterioration in the ability to detect an achromatic grapheme when the concurrent matched the background color. However, because this study predicted an impairment (as opposed to an enhancement), their task is potentially more susceptible to demand characteristics. We note that the experimenter in the present study was not blind to the experimental condition. However, the principal effect (if any) of lack of blindness would be to amplify demand characteristics, which we have argued are less likely to apply in our study than in Cohen Kadosh et al. (2009). The combination of a strong behavioral effect in Cohen Kadosh et al., and none at all in our study, is most simply explained by subjects responding according to how they believe they should, without hypnotically-induced alterations in perceptual abilities. This claim is entirely consistent with subjects in both studies having subjectively compelling experiences.

It is important to note that the ability to respond to the synesthesia suggestion was very variable across our participants. This is perhaps not surprising since perceptual hallucinations are difficult to evoke even in highly hypnotizable subjects (Bowers, 1998). It should also be noted that many developmental grapheme-color synesthetes fail to report colors during this task, at least during brief (1 s) presentations of the array (Ward et al., 2010). The strength of subjective experience of the colors was comparable to that of synesthetes. Anecdotally, some of our participants noted that the hypnotically suggested colors appeared to diminish over time. To reduce the impact of this, the suggestion was reinforced between blocks to sustain the colors but several participants struggled to maintain the suggestion all the same. Future research should combine extensive training of grapheme-color pairings (e.g., Rothen et al., 2011) with subsequent hypnotic suggestion.

If (at least some) of our hypnotized participants reported color experiences then why didn’t this help them to detect the embedded figure? One possibility suggested by our results is that the color hallucinations are primarily added after grapheme (and global figure) identification or, relatedly, that the “task” of adding color visualizations competes with the primary task of finding the embedded figure. This is supported by the analysis in which participants were divided (by median split) according to whether they reported many or few color visualizations. For the half of participants who experienced many colors, significantly more vivid colors were reported for accurate compared to inaccurate trials. This difference was not evident in the group who did not experience much color, perhaps due to a floor effect given that so little color was reported by these participants. The enhanced color experience for correct compared to incorrect trials for those who did experience color may reflect the ease with which the colors could be projected onto the graphemes by the participant. In this view, once the shape has been detected, the identity of digits within the array can more easily be inferred, potentially allowing easier visualization of the spatial localization of the red and green colors. In trials in which the shape has not been detected, the participant is performing two tasks at once; the conscious task of identifying the shape and the “unconscious” task of projecting colors onto black graphemes. The process of binding the grapheme with the concurrent color does not seem to occur as automatically in hypnotically suggested synesthesia, as compared to developmental synesthesia. Indeed, there is evidence that many hypnotic responses take up capacity by virtue of being hypnotic (Hilgard, 1986; Tobis and Kihlstrom, 2010; Wyzenbeek and Bryant, 2012; contrast Woody and Bowers, 1994). Developmental and artificially induced variants of synesthesia (i.e., hypnotic or drug induced) may be different. Auvray and Farina (in press) have explored this issue, and using their characterization of developmental synesthesia (as satisfying the criteria of the pairing of an inducer with a conscious concurrent, the idiosyncratic nature of the concurrent, and the concurrent being produced automatically and consistently) they have suggested hypnotic synesthesia satisfies the requirements of having a concurrent paired with an inducer, in an idiosyncratic and automatic way, but that consistency requires further investigation. Further, they suggest that the concurrent may be produced by imagery. Our results support a limited similarity between developmental and hypnotic synesthesia, and showing that despite the phenomenology, the concurrent may not be automatically produced (as shown by a lack of behavioral improvement).

How is it possible that our participants were able to generate color experiences at all (assuming, that is, that their subjective reports had some basis)? One possibility is that it relies on mechanisms normally used to support visual imagery. However, we did not assess this directly in our research. Other research suggests that there are individual differences between high and low hypnotizable subjects in the tendency to employ imagery in suitable contexts (e.g., Tellegen and Atkinson, 1974; Hilgard, 1979; Wilson and Barber, 1982; Roche and McConkey, 1990; Lynn and Green, 2011). However, the tendency to employ imagery in certain contexts may reflect strategic differences rather than ability differences given that high susceptible participants are not especially quick at visual information processing (Acosta and Crawford, 1985; Friedman et al., 1987) and are not especially high in rated imagery vividness (Jamieson and Sheehan, 2002; though compare the feats of imagery achieved by high but not low susceptible subjects in Mazzoni et al., 2009). These suggestions are tentative given that we did not run low hypnotizable subjects. Further, measuring the mental imagery abilities of participants would help clarify to what extent participants are able to use mental imagery to complete the tasks and how this relates to the individual profiles of hypnotic suggestibility (Cardeña, 2005; Terhune and Cardeña, 2010).

The lack of behavioral advantage for hypnotic synesthetes can be taken as evidence against functional similarity between hypnotic and natural synesthesia, but by the same token it provides support for the Cold Control theory of hypnosis (Dienes and Perner, 2007), and the class of theories which postulate no special ability is gained when an action is performed hypnotically (e.g., Kirsch and Lynn, 1999). Cold Control theory states that the subjective lack of volition in hypnosis is due to not forming the higher order thought (HOT) linked to the intention. In this sense, someone who responds to the suggestion “lift your arm” could lift their arm but not have the HOT “I am lifting my arm”. If this theory holds, then participants should not be able to perform better in the hypnosis block then they do in the control block. Our data indeed support this inference. Theories that postulate that hypnotic hallucination is perception-like in a way that goes beyond normal imagery (e.g., Brown and Oakley, 2004) are challenged by the current results.

In summary, hypnosis can induce verbal reports of phenomenological experience of grapheme-color synesthesia similar to those provided by developmental grapheme-color synesthetes, when applied in high susceptible participants. However, even though there are strong similarities in the subjective reports of natural and hypnotic synesthetes, this in not reflected in behavioral similarities. Highly hypnotizable subjects do not gain any perpetual abilities with a hypnotic suggestion that they did not have prior to hypnotic induction (Dienes and Perner, 2007).

## Author contributions

All authors (Hazel P. Anderson, Anil K. Seth, Zoltan Dienes, and Jamie Ward) contributed to the experiment design, analysis and write up. Hazel Anderson conducted the study.

## Conflict of interest statement

The authors declare that the research was conducted in the absence of any commercial or financial relationships that could be construed as a potential conflict of interest.

## Footnotes

^1^In their original study there were always three kinds of graphemes presented, but the test is commonly illustrated by the example of 5 s and 2 s.
